# Analysis of processes and costs of alternative packaging options of sterile goods in hospitals – a case study in two German hospitals

**DOI:** 10.1186/s13561-018-0218-2

**Published:** 2019-01-17

**Authors:** Markus Krohn, Josefine Fengler, Thomas Mickley, Steffen Flessa

**Affiliations:** Research Assistant, Chair of General Business Administration and Health Care Management, Faculty of Law and Economics, University of Greifswald, Friedrich-Loeffler-Straße 70, 17489 Greifswald, Germany

**Keywords:** Non-woven sterilisation wrap, Sterilisation container, Process analysis, Cost analysis, Time study, CSSD, OR, Monte-Carlo-simulation

## Abstract

**Background:**

Hospitals should monitor the costs of all direct and indirect processes in order to achieve efficiency and safeguard financial sustainability. One neglected process with significant costs is the processing of reusable medical devices and their packaging performed in the central sterilisation supply department and the operating room. The objective of this research is to analyse and compare processes and costs of four different packing alternatives, i.e. non-woven sterilisation wrap with two sheets, one-step wrap, sterilisation container with inner wrap and sterilisation container without inner wrap.

**Methods:**

We defined sub-processes that are directly related to the packaging options and measured them through a comprehensive time study. For all sub-processes and the total processes a distribution fitting and a Monte-Carlo-Simulation were performed. We calculated the costs for all sub-processes, i.e., costs for personnel, variable costs and the respective share of fixed and jump-fixed costs (e.g. depreciation of containers) associated with each packaging option. All results are discussed through various scenarios to evaluate the advantageousness of all packaging options.

**Results:**

The four packaging options are associated with different costs. “Sterile container without inner wrap” causes 2.05€ per use. The options “sterile container with inner wrap” (3.24€), “one-step sterilisation wrap” (3.44€) and “two sheets sterilisation wrap” (3.87€) cause higher costs. With regard to personnel costs the option “sterile container without inner wrap” clearly causes the lowest costs. In addition, variable costs are lower in case of sterile container. Sterile container only cause higher costs in the aspect of fixed and jump-fixed costs per packaging.

**Conclusions:**

The analysis shows that even under a broad set of scenarios the “sterile container without inner wrap” is the most cost-effective alternative. The evaluation of the options “sterile container with inner wrap” and “one-step sterilisation wrap” remains particularly interesting as they often yield comparable results. Both options cause approximately the same personnel costs, so the decision appears to be more dependent on the material prices for wrap or the frequency and duration of use for container. It turns out that the personnel time and consequently the personnel costs significantly influence the rational choice of the packaging options.

## Introduction

Since diagnosis-related groups were introduced in German hospitals (G-DRG) as a compulsory financing scheme in 2003, the management of these institutions has to assess the financial impact of all components of in-patient care. Before, hospitals could claim a refund from the health insurance funds for their actual expenditure with higher costs resulting in higher revenues. At that time, German hospitals could hardly generate a deficit and cost containment was rather irrelevant [1]. After a period of adjustment from 2003 to 2009, German hospitals now receive a DRG-based revenue for treating a patient which is – within a DRG-specific range between a lower and an upper time bound covering the vast majority of patients – independent from the length of stay and the actual costs of treatment [1]. Consequently, higher costs lead to a deficit in a way that hospital managers are forced to monitor the costs that are associated with in-patient cases much more than before [2]. A crucial instrument of cost containment are clinical pathways.

A clinical pathway is a series of interdependent processes and respective sub-processes which are required to treat a patient from admission to discharge. They are developed primarily in order to increase the effectiveness and quality of medical treatment. In addition, clinical pathways are proven instruments to reduce costs and increase cost-effectiveness of health care delivery. [3] For this purpose, all sub-processes which contribute to the treatment process and the costs of service delivery must be included in the analysis. This regularly includes service processes beyond the line of visibility from the perspective of the patient such as laboratory services or logistics [4].

One of these service processes which is not perceived by the patient and usually outside the focus of hospital managers is the reprocessing of medical devices in the Central Sterilisation Supply Department (CSSD). It is obvious that the provision of sterile materials by the CSSD is of high relevance for the quality of the entire treatment process [5]. At the same time, it significantly contributes to the total process costs [6–10]. According to the official handbook of calculating the cost per DRG, the cost of supplying sterile goods is included in the cost category “medical infrastructure”. It can be stated that the CSSD is one of the main cost drivers in this cost category, and the respective costs vary widely between (surgical) DRGs. However, our knowledge of the costs associated with these services is limited. In particular, there is hardly any literature on the costs of different packaging and processing options within the CSSD, especially sterilisation container with inner wrap (SCW), sterilisation container without inner wrap (SC), two sheets non-woven sterilisation wrap (TSW) as well as one-step non-woven sterilisation wrap (OSW). For a hospital manager it is crucial to know the costs of each of these alternatives in order to support the strategic decision on the packaging and processing option to perform in the hospital. However, no detailed cost analysis is known in the literature and no decision-support for best practice packaging options can be found so far.

This paper intends to close this research gap by analysing the processes and resource consumption of different packaging options of sterile goods in CSSD sub-processes. In the next section we present background information on different packaging systems in CSSDs. Afterwards the methodology of this study is presented consisting of a detailed process analysis of the entire sterile supply cycle, a comprehensive time study and the financial assessment. As the supply cycle does not only comprise CSSD, the processes of the operating room (OR) are included in the analysis. The fourth section will present the results. The analysis does not only present the processes and the respective resource consumption, but also compares sub-processes of the four main process options. The paper closes with a discussion of the results by presenting various scenarios and provides recommendations for hospital managers which are relevant not only to German health care providers.

### Packaging options

The supply and disposal of sterile goods is of high importance for maintaining functional services in hospitals, in particular for surgical units [11, 12]. Above all, it is crucial that sterilised materials (e.g. surgical instruments) maintain sterility until they are used at a later time and another place in the hospital. For this purpose, sterilised materials are professionally packaged as described in DIN EN ISO 11607-1 [13]. Two principal systems exist for wrapping of standard surgical instrument sets: non-woven sterilisation wrap and sterilisation container.

The non-woven sterilisation wrap is a one-way material covering the sterilised set of instruments or other products (e.g. implants, screws etc.). After the set is used, the wrap is disposed. Different types and qualities of non-woven sterilisation wrap are available on the market and have to be chosen based on the size and weight of the set. Typical wrap qualities range from about 70 g to 140 g per m^2^ for two layers of wrap. Further distinction can be made between a sequential wrap (two sheets of wrap per set) or a one-step wrap made of two sheets [10]. Sterilisation containers are made for frequent re-use and can be used over a wide range of set weights. The total container weight is limited by the sterilisation method used and the respective country’s working conditions act [14].

Wrapping and container packaging can be combined resulting in the before mentioned packing alternatives used in Europe and Northern America:

- two sheets non-woven sterilisation wrap (TSW): This is the traditional way of wrapping sterilised sets. The set is wrapped with one sheet of wrap. Afterwards it is wrapped again in another sheet. It is important to note that the two wrapping processes must be separated, i.e., it is not common practice to wrap a set within two sheets in a single step unless a special one-step wrap is used.
- one-step non-woven sterilisation wrap (OSW): A special wrap is used so that the set can be wrapped in one step. However, it requires a material consisting of two pieces of wrap specially attached to each other.
- sterilisation container with inner wrap (SCW): A sterilisation container with single layer wrapped surgical sets inside the container is commonly used in Germany. Lower quality wrap material is typically used in this case.
- sterilisation container without inner wrap (SC): According to DIN EN ISO 11607-1 container are rigid sterile barrier systems and thus inner wrapping in a sterile container is not obligatory so most hospitals worldwide (also some hospitals in Germany) use sterilisation containers without additional wraps.

All four alternatives are used in Europe and North America and show advantages and disadvantages. There are discussions on the sterility of the packaging during the entire logistic process. However, compliance with DIN EN ISO 11607-1 is normally proven by all manufacturers for all four alternatives. Thus, we assume that all necessary basic functions especially sterility up to the point of use can be taken for granted if one can ensure compliance with the instructions for use. In other words: In absence of evidence from the literature, this paper assumes that the quality of packaging is similar for all four systems. However, the choice of the sterile barrier system has a strong influence on the different sub-processes of the sterile supply cycle. In particular the following differences occur:

- Instrument cycle: A sterile container is part of every single step of the surgical instrument cycle, i.e. (1) disassembling and pre-cleaning, (2) cleaning and disinfection, (3) inspection and maintenance, (4) preparation for sterilisation, (5) sterilisation, (6) transport and storage, (7) use and (8) transport back to the CSSD. The use of surgical wrap starts with step (4) and ends with step (7). In addition, it needs a disposal sub-process after (7) not required by containers [11].
- Reprocessing: Most containers are made of anodised aluminum and have to be re-processed under special conditions separate from surgical instruments.
- Space requirement: The containers are voluminous, i.e., containers will need more space than wraps in particular for special reprocessing. This is especially challenging if hospitals are facing space constraints.
- Waste: As wraps are one-way materials they lead to a high volume of waste. This induces costs and environmental challenges. Some manufacturers have established recycling programs but still recycling costs and the associated challenges are much higher for wraps than for containers [15].
- Capital and running expenditure: Sterilisation container are investment goods (except of single use filters and locks depending on the container series used), wraps are disposables. Thus, using containers entails high but unique investment costs which are usually written-off in 8–10 years. Using sterilisation wrap incurs a steady flow of funds for consumables. The variable cost per sterilisation set is constant for the wrap but decreases for the container solution if the work load increases. Wraps bear the risk or chance of increasing or decreasing future material prices.

The differences between the systems shown are obvious. However, no detailed analysis of the economic dimension of the packaging system can be found in the literature. This paper focusses on the resource consumption and the respective costs of the four different packaging systems described above while assuming that the quality of sterility is identical for the respective systems.

## Methods

### Processes and materials

In order to analyse each packaging option, process information was required and therefore two German CSSDs were chosen that primarily produce sterile goods for maximum care hospitals. While the first CSSD mainly uses non-woven sterilisation wrap for the transport, reprocessing and storage of sterile goods, the second CSSD mainly uses sterilisation containers for this purpose. As a first step, main processes of the sterile supply cycle were defined. Due to the fact that main processes in hospitals take place at multiple locations and multiple professional groups contribute to the workflow, various department locations were taken as a basis for identifying main processes. In particular, processes of CSSD and OR were strictly differentiated.

As a second step, the main processes were subdivided into sub-processes thus achieving a more precise subsequent allocation of costs. For this purpose a team of researchers observed and documented all processes and sub-processes for several days in both hospitals. In addition, interviews with the leadership of the respective departments and other CSSD experts were conducted. Furthermore, the results of a literature analysis were included in the process analysis of the CSSDs and ORs. This analysis included the review of standard operating procedures of those particular institutions as well as DIN EN ISO standards and German laws related to the reprocessing of medical devices. The decisive factor for determining sub-processes were those procedure steps that required personnel and time for producing sterile instrument trays while peel packed instruments were not part of the analysis. Interim results of defining processes were discussed in several workshops with key-informants in terms of sterilisation and supply of medical devices.

As a third step, we determined the time consumption per service unit for each sub-process by measuring the time based on a so-called “elapsed time measurement”. [16] Consequently, the observers were present throughout the entire survey period and noted their observations continuously in the data entry form. Stopwatches were used, which means that timestamps were applied to calculate sub-process times retrospectively [17]. The overall time measurement included four observers who ascertained sub-process times parallel for a total of about 320 working hours per hospital. The main packaging options in the CSSDs were observed in equal shares. The results of the process analyses and the time measurement with in total 19,661 measured times are described in detail in the results section. An overview of the measured times per sub-process can be found in Table 4.

### Infrastructure adjustment

The total process times of all sub-processes of the entire sterile supply cycle is equal to the minimum overall time which one single instrument tray needs to pass through the CSSD and OR. This alone is an important information, but it does not allow to compare the different packaging alternatives. This is due to the fact that the infrastructure of both hospitals is slightly different which might bias the results. For this reason, only selected sub-processes were considered for further analysis. We focus only on those sub-processes that differ strongly between packaging options. Furthermore, we neglect sub-processes that are associated with transporting sterile goods. Consequently, the relevant sub-processes for a container comprise the packaging activities, the preparation of containers at the CSSD as well as the opening and closing activities in the OR. The respective sub-processes of non-woven sterilisation wrap consist of packaging activities, the additional process for wrap disposal at the CSSD and OR sub-processes. With regard to the chosen focus of one single reprocessed unit, we adjusted collected sub-process times belonging to multiple units. This was necessary to ensure that personnel times and costs would not be overestimated when multiple objects were processed at a time. For those cases sub-process times were divided through the amount of objects, e.g. multiple containers at one loading trolley.

### Further analysis

In order to determine a distribution of time consumption and costs of the alternatives it was necessary to perform a distribution fitting and a simulation. In a first step, outliers were identified by elimination of all process times outside the range of the fourfold standard deviation under and above the average. The methodology of elimination of outliers is based on the Chebyshev’s theorem which states that for unknown distributions at least 93.75% of all data is situated between the two bounds of four standard deviations in both directions from the mean value [18]. In sum 74 process times (0.38%) had to be eliminated. Furthermore, a four-character-acronym is defined for each process. The first character defines the process option (“W” = sterilisation wrap; “C” = sterilisation container), the second and third character is defined as a consecutive number (“01” up to “17”) and the fourth character defines the process location (“S” = CSSD; “O” = operation theatre).

To calculate the theoretical distribution behind the gained data the software tool EasyFit Professional Version 5.6 by MathWave Technologies was used. A lower bound of zero and an unknown upper bound were assumed in order to test all input samples to fit on the following distributions: Beta, Johnson SB, Kumaraswamy, Pert, Power Function, Reciprocal, Triangular, Uniform, Burr, Chi-Quadrat, Dagum, Erlang, Exponential, Fatigue Life, Frechet, Gamma, Generalized Gamma, Inverse Gaussian, Levy, Log-Gamma, Log-Logistic, Lognormal, Nakagami, Pareto, Pareto 2, Pearson 5, Pearson 6, Rayleigh, Rice, Weibull, Generalized Extreme Value, Generalized Logistic, Generalized Pareto, Log-Pearson 3, Phased Bi-Exponential, Phased Bi-Weibull and Wakeby.

**Fig. 1:**
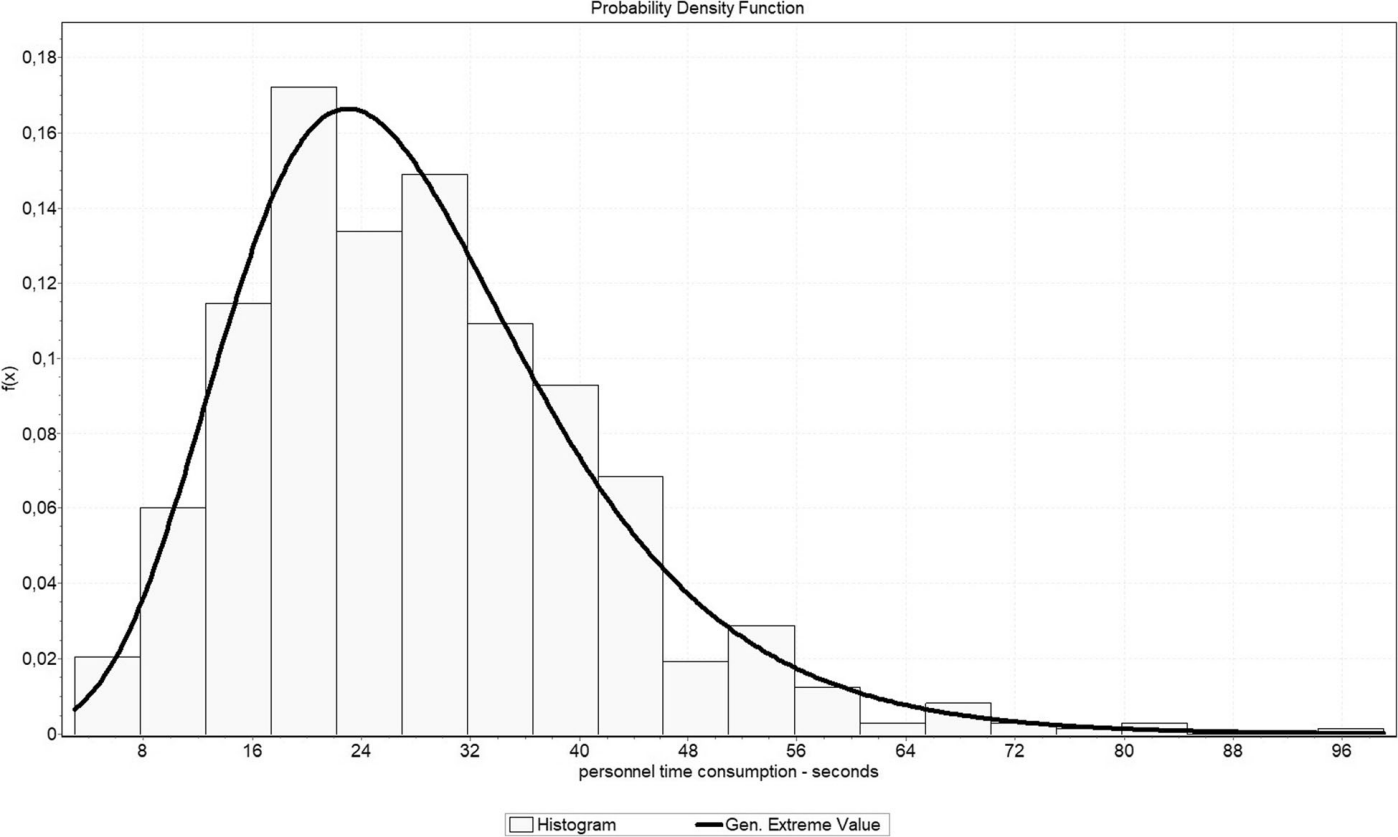
Probability Density function of process “bring, check und prepare the wrap (W04S)”

To decide which distribution fits best the Anderson-Darling (AD) test was used as defined in EasyFit Professional 5.6. Subsequently, the best-ranked distribution based on the AD-test was chosen for further analysis. Significant results at a = 0.01 based on AD-testing are marked with “*”. Furthermore, “°” shows significant distributions based on the Kolmogorov-Smirnov (KS) test. Due to the circumstances of some sub-processes being quite short (range of a few seconds) and the time study only measuring integer second-values, some distributions do not generate significant results. Consequently, the P-P-plot was also checked in order to decide if the highest ranked distribution could be used for simulation. As an example Fig. 1 shows the probability density function of process “bring, check und prepare the wrap (W04S)” by displaying the highest ranked distribution (Generalized Extreme Value) and the density function of the sample.

Based on the fitted distributions a Monte Carlo simulation was performed. In this case, 1000 random numbers were taken from the distribution of each sub-process. The results of individual simulation runs were combined in an additive manner, so as a result a determination of the overall distribution for the CSSD, the OR and the entire process was possible. The following assumptions had to be made in the context of the four defined procedural options. All assumptions can be regarded to as a correction factor of the process time, as some collected sub-processes are only partially allocable to the overall process. Table 1 shows the weighting of the individual sub-processes in case this value is less than one. A value below one means that the sub-process is not completely integrated into the overall process. By taking these correction factors into account, a total distribution of the CSSD, the OR and both areas was determined.

**Table 1:** Correction factors for sub-processes

Correction factor	For sub-process	Reason
0	bring, check and prepare the Containers with inner wrap (C07S) for SC	not part of total process in case of SC
0	cover tray with inner wrap, add label (C09S) for SC	not part of total process in case of SC
0	check and open Containers inner wrap (C13O) for SC	not part of total process in case of SC
0	dispose of inner wrap (C15O) for SC	not part of total process in case of SC
0	cover tray with first wrap layer, add label (W05S) for OSW	not part of total process in case of OSW
0.05840708	dispose of all collected wrap (W02S) for OSW and TSW	process has to be performed once per 17.12 produced units
0.05840708	provide new garbage bag (W03S) for OSW and TSW	process has to be performed once per 17.12 produced units
0.5	remove and dispose of wrap (W01S) for OSW	Assumption: half process time while using OSW
0.5	check and open wrapped tray (W08O) for OSW	Assumption: half process time while using OSW

An example of a process with a weight of less than one is “dispose of all collected wrap” (W02S) for OSW and TSW. The correction factor results from the ratio of the process “remove and dispose wrap” (W01S) and the process “dispose of all collected wrap” (W02S). While process W01S was measured 1360 times, process W02S could be measured 66 times. That results that process W02S has to be performed once per 17.12 units and the correction factor is 0.05840708.

### Costs and prices

In addition, staff costs per minute as well as costs for consumables and allocated costs were calculated. Table 2 shows the basic parameters of the respective calculations.

**Table 2:** Input parameters

Category	Parameter	Dimension	Value
Personnel	Working days per year (assuming 52 weekends, 30 days of vacation days, 10 holidays, 17 days of sickness, 5 days of further education and training)	days	199
	Maximum utilisation of staff	%	85
	Working time per day	hours	7.8
	Gross employer’s salary CSSD per year	€	36,000
	Gross employer’s salary OR per year	€	47,500
Variable cost	Wrap (one layer) for TSW	€	0.61
	One-Step-Wrap for OSW	€	1.27
	Inner Wrap for SCW	€	0.55
	Trayliner	€	0.11
	Labels	€	0.05
	Container Filters (2x)	€	0.11
	Container Seals (2x)	€	0.17
	Tape with Indicator	€	0.04
Fixed and jump-fixed Costs	Container	€	476.00
	Transport basket	€	154.70
	Life time container and transport basket	years	10
	Turnover rate of container and transport basket p.a.		120
	Waste disposal per 120 l	€	4.54
	Water consumption per cycle for large-scale washing system	litre	380
	Water (per 1000 l)	€	8.38
	Power consumption per cycle for large-scale washing system	kWh	6
	Electricity (per kWh)	€	0.20
	Chemicals per cycle for large-scale washing system	litre	0.6
	Chemicals (per litre)	€	5.06
	Percentage container of total load	%	70

According to these assumptions, the respective minute rate are 0.45 € (CSSD) and 0.60 € (OR).

The variable costs include wrap, trayliner, labels, container filter, container seal and indicator tape. The price information is based on the considered units. Since prices are also subject to strong fluctuations, scenarios are formed in the discussion section of this article. Table 2 shows the basic assumptions based on hospital price information. All values include value added tax as hospitals in Germany are not given a tax-credit for VAT paid.

The acquisition costs of containers and transport baskets were attributed to the process by defining a lifetime and the annual turnover. Assuming a lifetime of ten years and a turnover rate of 120 per year, the total usage frequency is 1200. It should be noted that the life time and frequency of circulation are variable. As part of the sensitivity analysis in the discussion, alternative scenarios are formed.

Annual total maintenance costs for the containers were allocated according to the number of containers and the annual turnover. It should be noted that the resulting repair costs of 0.80 € per container per year are not attributable exclusively to wear and tear. The costs also include the repair of damages due to handling errors and can therefore be considered as conservative. For the waste disposal, the costs per litre are 0.04 €. The survey found that the volume of waste per one layer wrap is around 3.504 l. Consequently, a volume of 7.009 l is assumed for the disposal of two sheets (TSW). Since OSW also exists on two firmly connected layers, we also assume 7.009 l. As part of SCW, only one sheet of trash is produced. Disposal costs for container filters and container seals are included in the area of variable costs.

Finally, the additional costs per unit produced were determined for the large-scale washing facility. The costs of the large-scale washing system result from the costs of a cleaning process divided by the load. Since, in addition to the containers, other materials are being cleaned in the large-capacity washing system, it is assumed that containers will take up 70% of the load per run. Thus, for the cleaning of containers, each run costs 5.19 €. Within the study, an average load of 22.45 containers per cycle was recorded. Due to the fact that the mean of loading was at 22.45 containers and the maximum loading was 33, the assumption that 70% of the costs are allocated to container cleaning can be considered as conservative.

Furthermore, it has to be taken into account that the options SCW and SC in certain circumstances require a higher capacity for container cleaning. In the base scenario, it is assumed that the use of containers does not result in full utilization of the large-capacity washing system. Acquisition, maintenance and repair costs are therefore not relevant to the decision. Two further scenarios are considered in the discussion. The first assumes that a larger large-capacity washing system will be needed to clean containers with additional acquisition costs of 17,500 €. Maintenance and repair costs are not relevant to the decision in this scenario, since it is assumed that the size of the machine has no influence on the absolute maintenance and repair costs. In the second scenario it is assumed that the CSSD requires an additional large-capacity washing system, which causes 167,500 € acquisition costs. Furthermore, 9500 € per year are taken into account for maintenance and repair. For the large-scale washing system a lifetime of 13.5 years and an annual production volume of 82.500 container is assumed. It should be noted that large-capacity washing systems are not used exclusively for cleaning containers. As previously stated, a value of 70% is assumed for the cleaning of containers and 30% for the cleaning of other products (for example, trolleys and large volume medical products).

## Results

### Process analysis

**Fig. 2:**
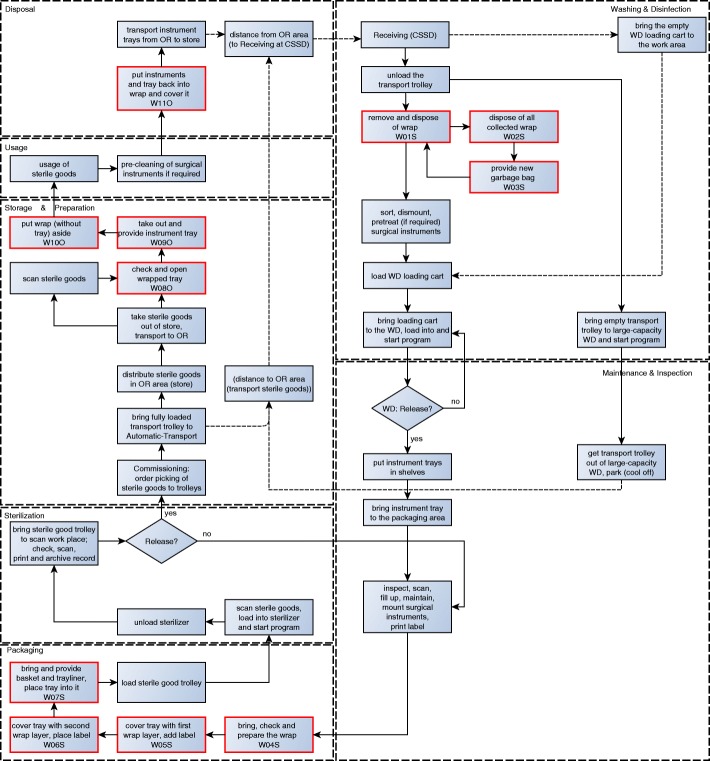
Sterile supply cycle of non-woven sterilisation wrap

**Fig. 3:**
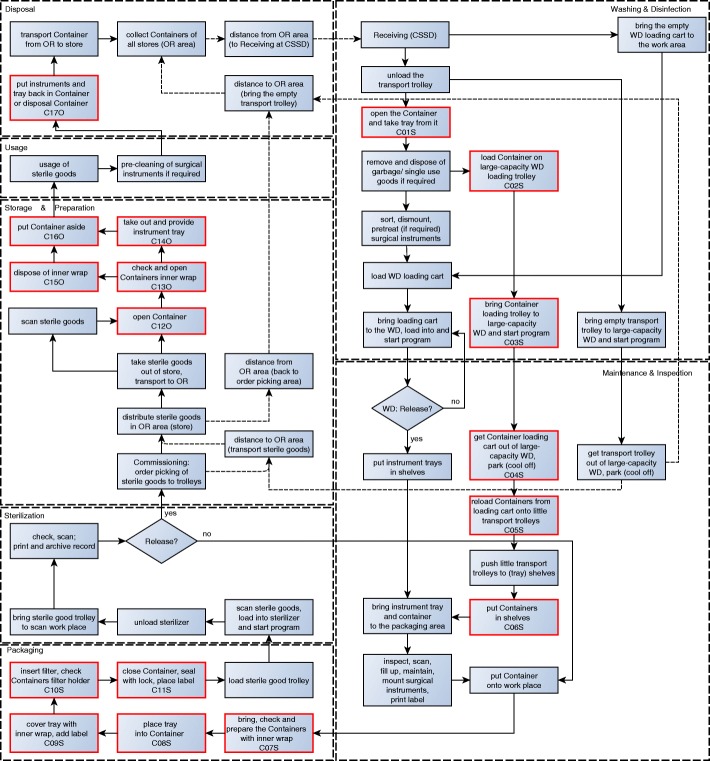
Sterile supply cycle of sterilisation container

As a result of observation, expert interviews, literature analysis and key-informant interviews we determined the processes as described in Figs. 2 and 3. In total 36 sub-processes for using non-woven sterilisation wrap (Fig. 2) and 48 sub-processes for using sterilisation container (Fig. 3) were identified (Table 3). With regard to the infrastructure adjustment, all sub-processes on which the analysis is based on, are noted in red in the process diagrams.

**Table 3:** Main processes and amount of sub-processes that were identified

Main process		Non-woven sterilisation wrap	Sterilisation container
CSSD	Washing and disinfection	10	11
	Maintenance and inspection	4	9
	Packaging	5	6
	Sterilisation	3	4
	Storage and preparation	9	11
OR	Usage	2	2
	Disposal	3	5
*Total*		*36*	*48*

Table 4 shows those processes which were included into the comparison of the alternatives. In total, the time of 19,661 sub-processes was measured by using stop-watches. Furthermore, the table shows the respective acronyms as well as the sample size from the time recording.

**Table 4:** Process definition and sample size

Process name	acronym	sample size
Open the Container and take tray from it	C01S	824
Load Container on large-capacity WD loading trolley	C02S	1101
Bring Container loading trolley to large-capacity WD and start program	C03S	2155
Remove and dispose of wrap	W01S	1124
Dispose of all collected wrap	W02S	65
Provide new garbage bag	W03S	66
Get Container loading cart out of large-capacity WD, park (cool off)	C04S	1998
Reload Containers from loading cart onto little transport trolleys	C05S	1139
Put Containers in shelves	C06S	1854
Bring, check and prepare the Containers with inner wrap	C07S	759
Bring, check und prepare the wrap	W04S	732
Place tray into Container	C08S	836
Cover tray with inner wrap, add label	C09S	810
Cover tray with first wrap layer, add label	W05S	1051
Insert filter, check Containers filter holder	C10S	661
Close Container, seal with lock, place label	C11S	812
Cover tray with second wrap layer, place label	W06S	1059
Bring and provide basket and trayliner, place tray into it	W07S	423
Open Container	C12O	253
Check and open Containers inner wrap	C13O	248
Check and open wrapped tray	W08O	262
Take out and provide instrument tray	C14O	285
Take out and provide instrument tray	W09O	239
Dispose of inner wrap	C15O	212
Put Container aside	C16O	211
Put instruments and tray back in Container or disposal Container	C17O	243
Put wrap (without tray) aside	W10O	104
Put instruments and tray back into wrap and cover it	W11O	135
Total		19,661

### Distribution fitting and Monte-Carlo-simulation

Table 5 shows the results of the distribution fitting. The corresponding parameters have been rounded off to four decimal places for reasons of better presentation. The distributions in combination with the correction factors form the basis for the Monte-Carlo-Simulation. It becomes clear that the theoretical distributions behind the sub-processes are manifold.

**Table 5:** Results of the distribution fitting of sub-processes

Process	Distribution	Parameters				
C01S	Wakeby	α = 10.1959	β = 0.0440	γ = 0	δ = 0	ξ = 0.8446
C02S	Johnson SB*	γ = 4.6286	δ = 2.4426	λ = 114.0524	ξ = −4.4557	
C03S	Log-Pearson 3	α = 13.6700	β = − 0.1317	γ = 3.2264		
W01S	Johnson SB*°	γ = 2.7028	δ = 1.4808	λ = 107.0346	ξ = −1.0275	
W02S	Beta*°	α_1_ = 0.7476	α_2_ = 1.1590	a = 0 (Fixed)	b = 173.0000	
W03S	Gen. Logistic*°	k = − 0.0314	σ = 5.0845	μ = 31.9906		
C04S	Pareto 2*	α = 0.5536	β = 0.0000			
C05S	Beta	α_1_ = 2.4664	α_2_ = 4.774	a = 0 (Fixed)	b = 35.3533	
C06S	Wakeby	α = 820.7058	β = 70.7089	γ = 11.5924	δ = −0.3908	ξ = −5.0945
C07S	Burr*	k = 0.6447	α = 4.0719	β = 9.2797		
W04S	Gen. Extreme Value*°	k = − 0.0385	σ = 10.6179	μ = 22.5843		
C08S	Pearson 6	α_1_ = 41.7009	α_2_ = 4.2773	β = 0.3206		
C09S	Log-Pearson 3*°	α = 63.1624	β = − 0.0679	γ = 7.7562		
W05S	Dagum*°	k = 1.1123	α = 3.8903	β = 35.8406		
C10S	Gen. Extreme Value*	k = 0.1449	σ = 3.8764	μ = 9.3659		
C11S	Inv. Gaussian*°	λ = 110.0178	μ = 34.5505			
W06S	Gen. Extreme Value*°	k = − 0.0255	σ = 28.8306	μ = 78.5859		
W07S	Dagum*°	k = 0.6310	α = 3.4634	β = 17.3266		
C12O	Log-Logistic*	α = 3.0702	β = 4.1318			
C13O	Log-Logistic*	α = 4.2215	β = 5.9365			
W08O	Burr*°	k = 0.6565	α = 4.4354	β = 14.3868		
C14O	Gen. Extreme Value	k = 0.3659	σ = 1.6206	μ = 3.6036		
W09O	Pearson 6*	α_1_ = 11.9523	α_2_ = 3.4768	β = 0.9310		
C15O	Burr*	k = 0.5800	α = 4.2272	β = 3.4437		
C16O	Log-Logistic*	α = 3.3153	β = 4.01482			
C17O	Johnson SB*°	γ = 1.5759	δ = 0.7162	λ = 81.6481	ξ = 1.4134	
W10O	Wakeby*°	α = 20.6865	β = 7.7626	γ = 4.8861	δ = −0.2130	ξ = 1.6206
W11O	Wakeby*°	α = 23.2417	β = 0.1569	γ = 0	δ = 0	ξ = 0.0504

*significant at a = 0,01 (AD-test)° significant at a = 0,01 (KS-test)

In order to answer the question of which distribution can be assumed for the CSSD, the OR and the overall process, the theoretical distribution behind the simulation data was determined. Table 6 shows that in the CSSD the processes for the options TSW, OSW and SCW follow a Generalized Extreme Value distribution. Option SC is subjected to a Johnson SB distribution. In the OR, the theoretic distributions are more diverse. These can be seen in Table 6 as well as the distributions of the total process (rounded at four decimal places).

**Table 6:** Results of the distribution fitting for the CSSD, the OR as well as the overall process based on the Monte-Carlo-Simulation of sub-processes

	location	distribution	parameters
TSW	CSSD	Gen. Extreme Value*°	k = −0,0873 σ = 40,9700 μ = 182,1558
	OR	Fatigue Life*°	α = 0,4057 β = 47,9642
	**Total**	Gen. Extreme Value*°	**k = −0,0659 σ = 44,4708 μ = 231,4989**
OSW	CSSD	Gen. Extreme Value*°	k = −0,0809 σ = 34,6168 μ = 135,9204
	OR	Johnson SB*°	γ = 27,767 δ = 15,727 λ = 221,3539 ξ = 59,964
	**Total**	Gen. Extreme Value*°	**k = −0,0804 σ = 38,4413 μ = 176,0546**
SCW	CSSD	Gen. Extreme Value*°	k = −0,1065 σ = 29,1803 μ = 140,0069
	OR	Wakeby*°	α = 424,5955 β = 42,8428 γ = 19,3004 δ = −0,06700 ξ = 14,2733
	**Total**	Lognormal*°	**σ = 0,1869 μ = 52,610**
SC	CSSD	Johnson SB*°	γ = 26,335 δ = 20,008 λ = 294,7783 ξ = 39,1941
	OR	Dagum*°	k = 62,639 α = 27,462 β = 11,5481
	**Total**	Fatigue Life*°	**α = 0,2133 β = 131,9283**

**Table 7:** Results of the Monte-Carlo-Simulation for the CSSD, the OR and the overall process – personnel time consumptions in seconds

	In seconds	CSSD	OR	*Total (by distribution)*	*Total as sum of CSSD and OR*
TSW	Mean	202.09	52.11	255.10	254.20
	Median	195.87	48.83	247.65	246.59
	Standard deviation	47.96	20.34	53.38	51.85
	Q1 (25%)	166.54	37.50	215.48	214.96
	Q3 (75%)	230.29	63.80	284.99	287.24
OSW	Mean	153.16	41.96	195.10	195.12
	Median	150.18	37.92	188.53	191.27
	Standard deviation	39.93	18.72	43.18	43.55
	Q1 (25%)	123.75	28.16	163.54	164.12
	Q3 (75%)	175.99	51.65	221.93	220.90
SCW	Mean	156.04	42.00	196.39	198.04
	Median	153.29	37.11	194.02	193.43
	Standard deviation	32.14	17.34	39.90	37.22
	Q1 (25%)	132.42	29.55	169.54	170.77
	Q3 (75%)	176.84	49.73	217.25	222.99
SC	Mean	104.50	30.23	135.23	134.73
	Median	101.22	25.17	131.58	129.82
	Standard deviation	24.96	18.25	29.34	30.85
	Q1 (25%)	86.08	19.22	113.58	111.76
	Q3 (75%)	120.16	34.60	153.85	153.72

Based on the distributions from Table 6, the personnel time consumption for all process options were again determined through 1000 simulation runs. In case of the CSSD these ranged between approximately 105 s and 202 s. It turns out that the option SC causes the lowest process times in the CSSD. TSW generates the highest expenditure with a process time that is around 92% higher. The remaining options OSW and SCW are nearly identical at 153 to 156 s, but the standard deviation of SCW is 20% lower. The OR results in the same ranking. While SC causes the least effort with about 30 s, the process with TSW takes about 52 s. Both OSW and SCW cause about 42 s, with almost identical standard deviations. The detailed results are shown in Table 7.

**Fig. 4:**
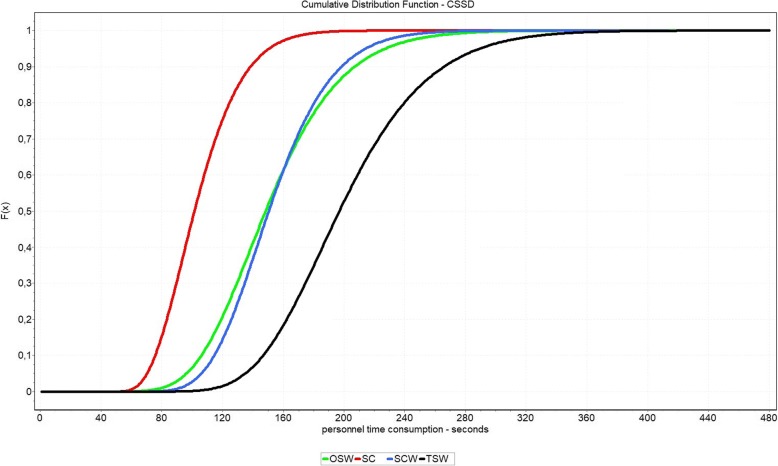
Cumulative Distribution Function of personnel time consumption in CSSD separated by packaging options

Figure 4 shows the aggregated density function of the process times of the CSSD personnel. It is clear that the SC option causes the lowest personnel costs and can be considered dominant in over 99.9% of cases. The TSW option is always dominated by all other options. Comparing the options OSW and SCW shows that OSW dominates the option SCW in about 61.99% of cases. However, the differences between the two options are considered very low. This can be exemplified by Q1 and Q3, where the difference is only about 4.2% or 1.1%.

**Fig. 5:**
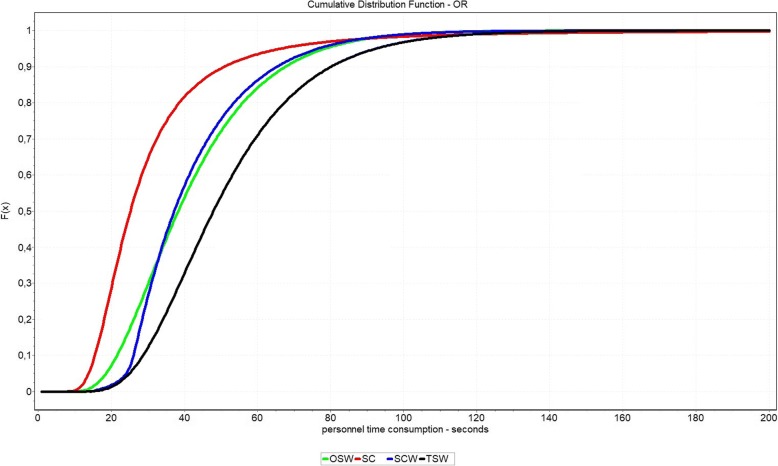
Cumulative Distribution Function of personnel time consumption in OR separated by packaging options

A similar picture can be observed in case of the processing times in the OR. According to the determined distribution, the option SC is dominant in about 97.7%. However, the OSW option dominates the SCW option in only about 37.33% of cases. At Q1, SCW causes about 7.1% higher process times than OSW, while OSW causes 4.6% higher times at Q3. The distribution function is shown in Fig. 5.

### Cost-analysis

The determination of the personnel costs per packaging option was carried out by formation of a minute set. As a result, average personnel costs amount to 2.05 € for TSW, 1.58 € for OSW, 1.60 € for SCW and 1.09 € for SC based on the times shown. The variable costs show a value of 1.42 € for TSW and a value of 1.46 € for the OSW option. The SCW option costs 0.87 € and the SC option 0.32 €.

In terms of additional costs, it becomes clear that the acquisition costs of a container cause a total of 0.40 € per use (Option SCW and SC). There is also a value of 0.01 € for repairs. The costs for the transport baskets are 0.13 € per use (Option TSW and OSW). Disposal costs in the context of TSW and OSW result at around 0.27 €. The disposal costs for SCW are around 0.13 €. The costs of the large-capacity washing maschine are 5.19 € per run. Within the study, an average load of 22.45 containers per cycle was recorded. Consequently, per container about 0.23 € are attributable (SCW and SC).

For the material costs as well as the allocated costs results show 1.81 € for TSW, 1.86 € for OSW, 1.64 € for SCW and 0.96 € for SC. In comparison to the personnel costs, a partially changed ranking shows up. The previously dominant option SC remains dominant. The option OSW (previously approximately equal to SCW) is now dominated by all other options.

Table 8 shows the total result of personnel and material costs. It turns out that SC shows the least costs at 2.05 €. Rank two takes the SCW option, which at 3.24 € causes approx. 58.0% higher costs in packaging-related processes. The costs of TSW are ranked third with 3.44 € (+ 67.5% compared to SC). OSW occupies fourth place, with costs of 3.87 €, which is about 88.5% higher than SC.

**Table 8:** Results of cost-analysis

Cost category		TSW	OSW	SCW	SC
Personnel Cost in €	CSSD	1.53	1.16	1.18	0.79
	OR	0.52	0.42	0.42	0.30
	**Total**	**2.05**	**1.58**	**1.60**	**1.09**
Material Costs in €	Wrap	1.23	1.27	0.55	–
	Trayliner	0.11	0.11	–	–
	Labels	0.05	0.05	0.05	0.05
	Container Filter	–	–	0.11	0.11
	Container Seal	–	–	0.17	0.17
	Tape with Indicator	0.04	0.04	–	–
	**Total**	**1.42**	**1.46**	**0.87**	**0.32**
Special Cost in €	Container Cost	–	–	0.40	0.40
	Transport Box	0.13	0.13	–	–
	Container Repair	–	–	0.01	0.01
	Waste Disposal	0.27	0.27	0.13	–
	Large-Capacity washing system	–	–	0.23	0.23
	**Total**	**0.39**	**0.39**	**0.77**	**0.64**
Total in €		**3.87**	**3.44**	**3.24**	**2.05**

**Fig. 6:**
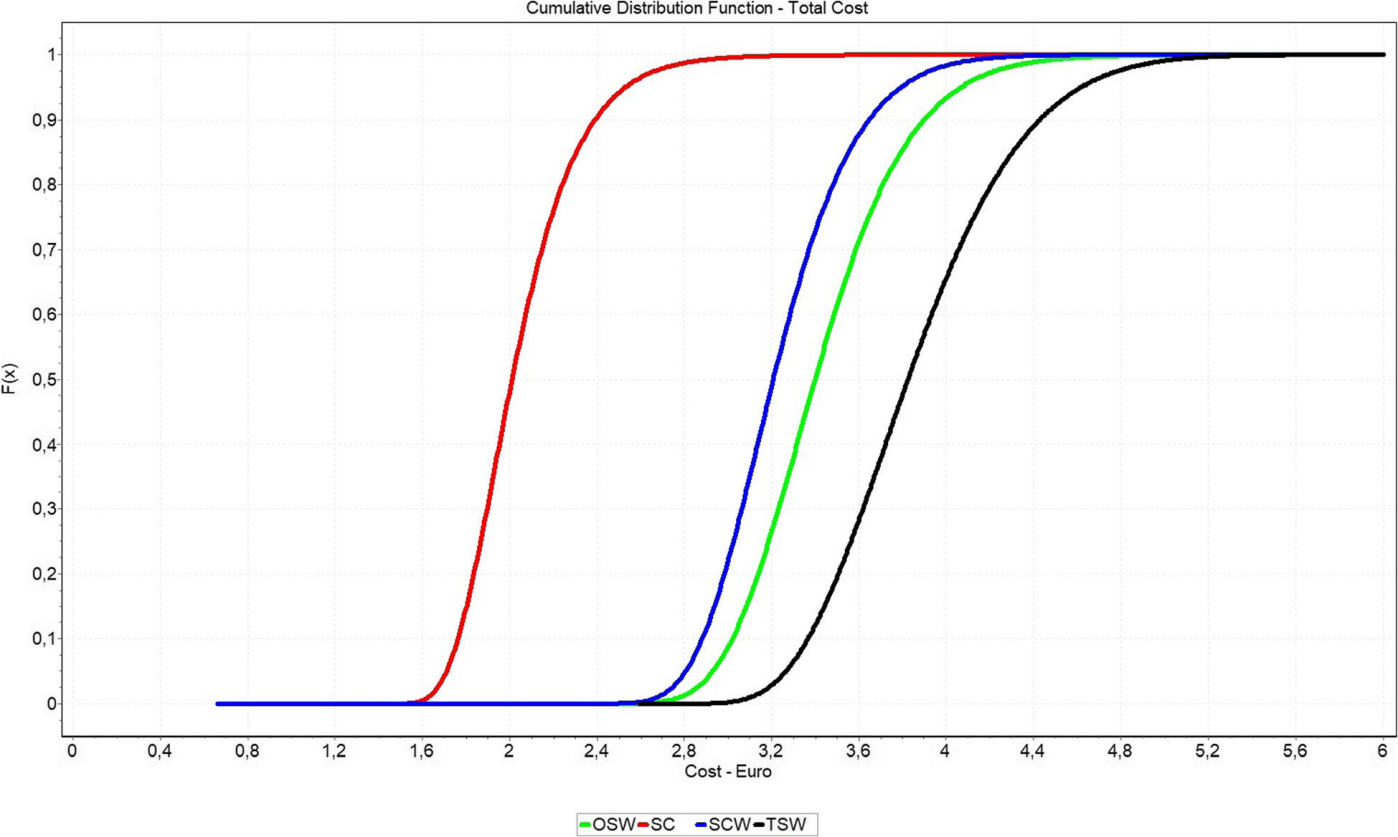
Cumulative Distribution Function of total cost separated by packaging options

Figure 6 combines the personnel costs of CSSD and OR based on the distributions. Furthermore, the figure includes the material costs and the transferred costs according to Table 8. This results in a Monte-Carlo-Simulation of the costs.

In the overall result we clearly see that the option SC dominates all other options. Rank two is now taken solely by SCW. The OSW option dominates the TSW option by approximately 100%. The significant right shift of the OSW option is caused by the material costs of this option. Based on the presented input values, the following sequence of options can be displayed.

- Rank 1 - SC (Sterile Container without inner wrap) - mean: 2.05 €
- Rank 2 - SCW (Sterile Container with inner wrap) - mean: 3.24 €
- Rank 3 - OSW (one-step non-woven sterile wrap) - mean: 3.44 €
- Rank 4 - TSW (two sheets non-woven sterile wrap) - mean: 3.87 €

## Discussion

The results of this analysis show that the SC option causes the least costs. For the decision maker however, the question arises how stable this result is if major assumptions are subject to change. Consequently, we have to assess the sensitivity of these results if major parameters change. The following 33 scenarios analyse the consequences of the following changes of parameters:

- Part A (personnel costs)
  - ○ staff costs increase by 10%
  - ○ staff costs increase by 20%
  - ○ personnel costs are not relevant to the decision
- Part B (material and special costs):
  - ○ material and special costs increase by 10%
  - ○ material and special costs increase by 20%
  - ○ material costs decrease by 10%
  - ○ material costs decrease by 20%
  - ○ acquisition costs for containers increase by 10%
  - ○ acquisition costs for containers increase by 20%
  - ○ acquisition costs for containers increase by 50%
  - ○ material cost for wrap decrease by 10%
  - ○ material cost for wrap decrease by 20%
  - ○ material cost for wrap decrease by 50%
  - ○ material cost for wrap (TSW and OSW) increase by 10%
  - ○ material cost for wrap (TSW and OSW) increase by 25%
  - ○ material cost for wrap (TSW and OSW) increase by 50%
- Part C (changes in usage)
  - ○ turnover rate of containers and transport baskets increases by 10%
  - ○ turnover rate of containers and transport baskets increases by 25%
  - ○ turnover rate of containers and transport baskets increases by 50%
  - ○ turnover rate of containers and transport baskets decreases by 10%
  - ○ turnover rate of containers and transport baskets decreases by 25%
  - ○ turnover rate of containers and transport baskets decreases by 50%
  - ○ transport baskets are not used
  - ○ containers are used only 5 years
  - ○ containers are used for 15 years
  - ○ containers are used for 20 years
  - ○ larger large-capacity washing system needed
  - ○ additional large-capacity washing system needed
- Part D (extreme scenarios)
  - ○ personnel costs are not relevant to the decision and material costs for wrap decrease by 75%
  - ○ material costs for wrap decrease by 50% and turnover rate of containers and transport baskets increases by 50%
  - ○ material costs for wrap decrease by 91% and transport baskets are not used
  - ○ additional large-capacity washing system needed, material costs for wrap decrease by 50% and transport baskets not used
  - ○ personnel costs not relevant to the decision, material costs for wrap decrease by 40%, transport baskets not used and additional large-capacity washing system needed

Part A: As part of the scenario building process, personnel costs were varied. The changes + 10% and + 20% can have two meanings. Either 10% or 20% increase in personnel costs or 10% and 20% increase in personnel time consumptions. It turns out that the order of the results does not change. The reason is that the ranking of the personnel time consumptions follows the overall ranking. Another situation arises when personnel costs are not relevant to the decision. This situation occurs when the differences in time consumptions do not require additional personnel. Consequently, this situation only occurs if – regardless of the packaging option – the staff is working within its capacity limits. In this scenario, TSW (now in rank three) costs less than OSW (now in rank four). In all scenarios, SC causes the least costs and SCW the second lowest costs. The changed values are shown in Table 9.

**Table 9:** Scenario analysis – Part A

Packaging Option	TSW		OSW		SCW		SC	
Scenario	Cost	Rank	Cost	Rank	Cost	Rank	Cost	Rank
Personnel costs + 10%	4.07 €	4	3.59 €	3	3.40 €	2	2.16 €	1
Personnel costs + 20%	4.28 €	4	3.75 €	3	3.56 €	2	2.27 €	1
Personnel costs not relevant	1.81 €	3	1.86 €	4	1.64 €	2	0.96 €	1

Part B: Within the scenarios, the material or contribution costs were varied. It turns out that a change in the variable costs by + 10% and + 20% and the special costs by + 10% and + 20%, does not result in any changes in the ranking. The same applies to the increase in container price by + 20%. Only if the container price is increased by + 50% is the option OSW beneficial compared to SCW (0.004€). An identical picture is found in the change in price of the sterilisation fleece. A change of - 20% will not influence the original ranking. Only if the price for wrap is reduced by 50% does the OSW option benefit SCW. TSW will not be advantageous over SCW. This is due to relatively high staff costs for TSW. Furthermore, the effects of a higher price for non-woven wrap in the TSW and OSW options were investigated. The reason for this is that with a higher weight of the sterile goods, expensive nonwovens must be used, while there are no changes in the container options. It turns out that with a 50% increase of prices for wrap, TSW and OSW options can cost more than twice as much as SC. In reality, large price fluctuations can exist. On one hand side, discounts for high sales volumes lead to strong benefits, on the other hand side, low sale volumes or high weights of the instrument tray (requiring stronger wrap) significantly affect the costs. Consequently, the price fluctuations assumed here are rather realistic. The result shows that the price of wrap (including disposal) is the relevant cost driver. In the baseline scenario, they cause about 38.6% of the packaging related costs in the TSW option. In the option OSW it is about 44.7% and in the option SCW about 21.0%. Table 10 displays the scenarios in detail.

**Table 10:** Scenario analysis – Part B

Packaging Option	TSW		OSW		SCW		SC	
Scenario	Cost	Rank	Cost	Rank	Cost	Rank	Cost	Rank
Material and special costs + 10%	4.05 €	4	3.62 €	3	3.40 €	2	2.15 €	1
Material and special costs + 20%	4.23 €	4	3.81 €	3	3.57 €	2	2.24 €	1
Material costs −10%	3.72 €	4	3.29 €	3	3.15 €	2	2.02 €	1
Material costs −20%	3.58 €	4	3.14 €	3	3.07 €	2	1.99 €	1
Price container + 10%	3.87 €	4	3.44 €	3	3.28 €	2	2.09 €	1
Price container + 20%	3.87 €	4	3.44 €	3	3.32 €	2	2.13 €	1
Price container + 50%	3.87 €	4	3.44 €	2	3.44 €	3	2.25 €	1
Material costs for wrap −10%	3.74 €	4	3.31 €	3	3.19 €	2	2.05 €	1
Material costs for wrap −20%	3.62 €	4	3.18 €	3	3.13 €	2	2.05 €	1
Material costs for wrap −50%	3.25 €	4	2.80 €	2	2.97 €	3	2.05 €	1
Material costs for wrap + 10% for TSW and OSW	3.99 €	4	3.56 €	3	3.24 €	2	2.05 €	1
Material costs for wrap + 25% for TSW and OSW	4.17 €	4	3.75 €	3	3.24 €	2	2.05 €	1
Material costs for wrap + 50% for TSW and OSW	4.48 €	4	4.07 €	3	3.24 €	2	2.05 €	1

Part C: The third part examines changes in usage. This includes changes in the turnover rate and the non-use of transport baskets in the TSW and OSW option. Furthermore, changes in the period of usage of containers is examined. It turns out that an increase in the turnover rate has no influence on the ranking of the results. The increased usage of containers and transport baskets, however, leads to a decrease in the packaging related costs per piece produced. Another result shows in case of the reduction of the turnover rate. While the results are unchanged at a − 25% reduction, a 50% reduction makes OSW less expensive than SCW. The reason for this are the acquisition costs for containers. With a lifetime of 10 years and a circulation of 60 per year, the containers are used only 600 times in total. This shows that the circulation rate has a significant impact on costs. Furthermore, the changes in the non-use of transport baskets were examined. It turns out that the option OSW now dominates the SCW. However, this does not take into account the additional measures required to store the sterile goods in order to compensate for the disadvantage of the missing transport baskets. Thus, the option will only be beneficial if the additional cost for storage is less than 0.04 € per piece. It should be noted that option SC is still dominant in this case. Finally, the influence of the usage time of the containers was examined. While an extension to 15 or 20 years increases the advantage of the SCW and SC options, in case of a reduction to a 5-year usage, the OSW option becomes dominant over SCW. However, it should be noted that a useful lifetime of 5 years with a circulation frequency of 120 per year is rather pessimistic. On the basis of surveys of 36 CSSDs in Germany, the average period of usage was 13.25 years, with a standard deviation of 6.86 years and a median of 12 years. It should be noted, that even with a usage time of only 5 years, the SC option is dominant. This is due to the low personnel time consumption of the option. As part of the large-capacity washing system scenarios, it becomes clear that a larger washing system has only a small impact on the costs per item (0.01 €). The reason for this is that the depreciation per year is very small, since only the difference of the acquisition costs and no maintenance and repair costs have to be considered. If an additional system is required, the costs of the SCW and SC options increase significantly. The cost of the SCW option is then almost identical to the OSW option. However, it turns out that the SC option still causes the least cost. Table 11 shows the scenarios in detail.

**Table 11:** Scenario analysis – Part C

Packaging Option	TSW		OSW		SCW		SC	
Scenario	Cost	Rank	Cost	Rank	Cost	Rank	Cost	Rank
Turnover rate (container and transport basket) + 10%	3.85 €	4	3.42 €	3	3.20 €	2	2.01 €	1
Turnover rate (container and transport basket) + 25	3.84 €	4	3.41 €	3	3.16 €	2	1.97 €	1
Turnover rate (container and transport basket) + 50%	3.82 €	4	3.39 €	3	3.11 €	2	1.92 €	1
Turnover rate (container and transport basket) -10%	3.88 €	4	3.45 €	3	3.29 €	2	2.10 €	1
Turnover rate (container and transport basket) -25%	3.91 €	4	3.48 €	3	3.38 €	2	2.19 €	1
Turnover rate (container and transport basket) -50%	4.00 €	4	3.56 €	2	3.65 €	3	2.46 €	1
Transport baskets not used	3.63 €	4	3.20 €	2	3.24 €	3	2.05 €	1
Container used for 5 years	3.87 €	4	3.44 €	2	3.64 €	3	2.45 €	1
Container used for 15 years	3.87 €	4	3.44 €	3	3.11 €	2	1.92 €	1
Container used for 20 years	3.87 €	4	3.44 €	3	3.04 €	2	1.85 €	1
Larger large-capacity washing system needed	3.87 €	4	3.44 €	3	2.25€	2	2.06€	1
Additional large-capacity washing system needed	3.87 €	4	3.44€	3	3.43 €	2	2.24 €	1

Part D: The last three scenarios can be regarded as extreme scenarios. It turns out that the replacement of the SC option as best option requires massive changes. The SC option becomes third-rate, if personnel costs are not relevant to the decision and the price for wraps decreases by 75%. If personnel costs are included, the option SC even remains optimal if the wrap price decreases by 50% and the turnover rate is reduced by 50%. The third scenario shows that the TSW option would only become dominant over the SC option by 0.01 € if the wrap price were to fall by 91% and transport baskets would not be used. It turns out that personnel costs have a massive impact on determining the best option. Scenario number four includes a 50% reduction in wrap price, the non-use of transport baskets and the need for an additional washing system for container use. Even in this scenario, the SC option dominates. The last extreme scenario shows under which circumstances both wrap options dominate the container options. If personnel costs are not relevant to the decision, the price for wrap is reduced by 40%, transport baskets are not used and an additional washing system for containers is required, the options TSW and OSW dominate. Table 12 displays the ranking of these five scenarios.

**Table 12:** Scenario analysis – Part D

Packaging Option	TSW		OSW		SCW		SC	
Scenario	Cost	Rank	Cost	Rank	Cost	Rank	Cost	Rank
Personnel costs not relevant and material costs wrap −75%	0.89 €	1	0.90 €	2	1.23 €	4	0.96 €	3
Material costs wrap −50% and turnover rate (container and transport basket) -50%	3.38 €	4	2.93 €	2	3.37 €	3	2.46 €	1
Material costs wrap −91% and transport baskets not used	2.51 €	3	2.04 €	1	2.74 €	4	2.05 €	2
Additional large-capacity washing system needed, wrap −50% and transport baskets not used	3.02 €	3	2.56 €	2	3.15 €	4	2.24 €	1
Personnel costs not relevant, material costs wrap −40%, transport baskets not used and additional large-capacity washing system needed	1.09 €	1	1.11 €	2	1.60 €	4	1.14 €	3

The scenarios underline that the option SC is dominant and favorable in almost all constellations. Furthermore, the option SCW causes the second lowest costs. Often the option SCW and OSW are close together. The reason for this is that the higher material costs of the OSW option are almost compensated by higher special costs (contribution costs for containers and large-capacity washing system). However, this does not apply if stronger and more expensive fleece must be used as protective packaging. In some cases significantly higher costs result in the option OSW. The option TSW causes the highest costs in most cases. This is due to the higher process times and thus higher personnel costs.

This study has a number of limitations. Firstly, it focuses purely on the economic dimension assuming that all the four options achieve the same level of hygiene-quality, which is in agreement with the fact that compliance with DIN EN ISO 11607-1 is normally proven by all manufacturers for all four alternatives. Meaning that we did not have to compare cost-effectiveness ratios but could concentrate on a cost-comparison alone. However, further research must follow to give more evidence to this assumption.

Secondly, our study calculates the actual costs based on the real-life situation in two hospitals. We do not assess whether the processes are efficient but assume that the time consumption reflect the real situation. It might be worthwhile to produce a standard-costing for “best” processes instead of “actual” processes. Thirdly, the analysis took a strategic perspective, i.e., we assume that all costs are relevant. If we take a short-term perspective, depreciation charges are to be neglected as they are sunk costs in the short-run. This will not change the ranking of the alternatives, but has an impact on the comparative advantages of the options container versus wrap. Finally, our analysis is based on two German hospitals with respective laws and regulations, personnel costs, processes and financing systems.

## Conclusion

This analysis shows that the costs of different packaging alternatives do differ – a result of high relevance to hospital managers worldwide. This result is not a consequence of structural differences of the two hospitals analysed as we concentrated only on those processes which are comparable. Instead, differences in time and costs are consequences of the packaging alternatives. However, this result should not be overestimated. Instead, each CSSD or hospital should analyse its own situation, requirements and circumstances. For some institutions, for instance, capital is the scarcest agent of production as these institutions have no access to loans while relying on (declining) government funded grants to purchase containers. For these institutions, leasing of containers or using wraps might be the most rational solution. For other institutions, rising costs of personnel or a shortage of personnel are the most pressing influencing factors. In this case, the alternative with the lowest consumption of personnel time should be sought. Although an advantage of about 98 s per sterilisation set (SC vs. TSW) appears low, this difference accumulates to more than one full-time position in the CSSD if we assume an annual output of 49,000 units. From the economic point of view, it becomes clear that factors such as personnel time consumptions, turnover rates, material costs and acquisition costs should always be taken into account.

It will be challenging to see whether an analysis in another country will reproduce the same results. Thus, this paper calls for more research in the economics of hospital management and in particular the economics of hygiene management.

## Abbreviations

€: Euro
AD: Anderson-Darling
CSSD: Central Sterilisation Supply Department
DIN: German Institute for Standardization
DRG: diagnosis-related groups
EN: European Committee for Standardization
G-DRG: German-diagnosis-related groups
ISO: International Organization for Standardization
KS: Kolmogorov-Smirnov
OR: operating room
OSW: one-step non-woven sterilisation wrap
Q: Quantile
SC: sterilisation container without inner wrap
SCW: sterilisation container with inner wrap
TSW: two sheets non-woven sterilisation wrap
VAT: value added tax
WD: washing device
